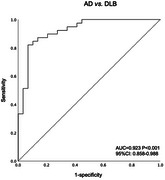# Neuropsychological Features for Differentiating Dementia with Lewy Bodies and Alzheimer's Disease

**DOI:** 10.1002/alz70857_102693

**Published:** 2025-12-25

**Authors:** Xin Ma, MING ZHANG, Yaonan Zheng, Xingyu Zhang, Mang Zhang, Yuhan Xie, Mengmeng Xia, Tao Li, Haifeng Zhang, Chun Tang, Xin Yu, Huali Wang

**Affiliations:** ^1^ Peking University Institute of Mental Health (Sixth Hospital), Beijing, Beijing, China; ^2^ The Third Affiliated Hospital of Sun Yat‐sen University, Guangzhou, China; ^3^ Peking University, Beijing, China; ^4^ Peking University Institute of Mental Health (Sixth Hospital), Beijing, China; ^5^ Peking University, Beijing, Beijing, China; ^6^ Beijing Jiahua Social Work Center, Beijing, Beijing, China; ^7^ Dementia Care and Research Center, Peking University Institute of Mental Health (Sixth Hospital), Beijing, China; ^8^ NHC Key Laboratory for Mental Disorders, Beijing, Beijing, China; ^9^ National Clinical Research Center for Mental Disorders, Beijing, China

## Abstract

**Background:**

Dementia with Lewy bodies (DLB) and Alzheimer's disease (AD) are neurodegenerative conditions that share overlapping cognitive features, making differentiation challenging. This study aimed to compare cognitive impairments between AD and DLB and identify a set of assessments to distinguish these diseases.

**Method:**

A total of 159 subjects (53 in each group: DLB, AD, and normal controls [NC]) participated in the study. All participants were administered tests from the Chinese Neuropsychological Normative (CN‐NORM) Project ‐ Consensus Battery (CNCB), which assess six cognitive domains. Cognitive variables were entered into a binary logistic regression model using forward stepwise selection to identify key tests that differentiate DLB from AD. The diagnostic accuracy of the final model was evaluated using receiver operating characteristic (ROC) curve analysis.

**Result:**

Both DLB and AD patients performed significantly worse than NC across all cognitive domains (*p* < 0.05). Specifically, DLB patients scored significantly higher on the Geriatric Depression Scale and the Geriatric Depression Inventory (GDI) compared to AD patients (*p* < 0.001). DLB patients also performed significantly worse than AD patients on attention tasks (*p* < 0.05), executive function tasks (*p* < 0.05), visuospatial tasks (*p* < 0.05), and social cognition tasks (*p* < 0.05). In contrast, AD patients showed poorer performance on memory tests, particularly the Hopkins Verbal Learning Test, Brief Visual Memory Test, and Logic Memory Test (*p* < 0.05). Logistic regression analysis indicated that scores on the GDI, Hong Kong Brief Cognitive Test, Digit Symbol Substitution Test, and Judgment of Line Orientation Test could effectively differentiate DLB from AD, achieving high diagnostic accuracy with an area under the curve (AUC) of 0.923 (95% CI: 0.858‐0.988, *p* < 0.001).

**Conclusion:**

This study identified distinct cognitive profiles for AD and DLB and developed a diagnostic model with high accuracy. These findings may provide a practical tool for improving differential diagnosis and guiding clinical decision‐making.